# Nitro-Glycerine in Migraine, Epilepsy and Anæmic Headaches

**Published:** 1881-12

**Authors:** 


### NITRO-GLYCERINE IN MIGRAINE, EPILEPSY AND ANÆMIC HEADACHES.

Dr. Wm. A. Hammond (*Virginia Med. Monthly*, Oct., 1881), publishes his experience with the use of nitro-glycerine in the above named affections. The form in which he employs the agent is to take the homœopathic decimal preparation, and dilute it with ninety parts of alcohol (℞ Nitro-glycerine, one-tenth, 40 minims, alcohol 6 drams), of this he gives in migraine *one drop* every fifteen minutes until relief is obtained. It has rarely been necessary to give a third dose in the attack. It is only applicable, however, in the anæmic form of migraine. In epilepsy, the remedy—of the same strength as above—is given in one drop doses three times a day, increasing the dose one drop every month.

In cases of anæmic headache, or vertigo, due to anæmia of the brain, the same remedy will frequently be successful.

Dr. Hammond concludes his interesting paper by cautioning those about to use the remedy to secure a pure preparation of known strength, and exercise proper care in its administration.

